# A Flexible, Large-Scale Sensing Array with Low-Power In-Sensor Intelligence

**DOI:** 10.34133/research.0497

**Published:** 2024-11-13

**Authors:** Zhangyu Xu, Fan Zhang, Erxuan Xie, Chao Hou, Liting Yin, Hanqing Liu, Mengfei Yin, Lang Yin, Xuejun Liu, YongAn Huang

**Affiliations:** ^1^ State Key Laboratory of Intelligent Manufacturing Equipment and Technology, Huazhong University of Science and Technology, Wuhan 430074, China.; ^2^ Flexible Electronics Research Center, Huazhong University of Science and Technology, Wuhan 430074, China.

## Abstract

Artificial intelligence of things systems equipped with flexible sensors can autonomously and intelligently detect the condition of the surroundings. However, current intelligent monitoring systems always rely on an external computer with the capability of machine learning rather than integrating it into the sensing device. The computer-assisted intelligent system is hampered by energy inefficiencies, privacy issues, and bandwidth restrictions. Here, a flexible, large-scale sensing array with the capability of low-power in-sensor intelligence based on a compression hypervector encoder is proposed for real-time recognition. The system with in-sensor intelligence can accommodate different individuals and learn new postures without additional computer processing. Both the communication bandwidth requirement and energy consumption of this system are significantly reduced by 1,024 and 500 times, respectively. The capability for in-sensor inference and learning eliminates the necessity to transmit raw data externally, thereby effectively addressing privacy concerns. Furthermore, the system possesses a rapid recognition speed (a few hundred milliseconds) and a high recognition accuracy (about 99%), comparing with support vector machine and other hyperdimensional computing methods. The research holds marked potential for applications in the integration of artificial intelligence of things and flexible electronics.

## Introduction

With the emergence of artificial intelligence (AI) and the internet of things (IoT), artificial intelligence of things (AIoT) systems enabled by sensor networks can intelligently perceive the condition of the surroundings and autonomously recognize them [1]. With unique mechanical and electrical characteristics [2–8], flexible sensors have been widely utilized in health monitoring [9–12], robot e-skin [13–16], human–computer interaction [17–19], flying perception [20–22], and other fields, serving as a crucial method for IoT to collect data. Large-area and flexible pressure sensing arrays can cover a greater detection area, thereby capturing more pressure information. This is crucial for applications requiring the monitoring or measurement of extensive pressure fields [23,24]. High-density sensing arrays enable simultaneous monitoring of multiple points, allowing for real-time detection of changes at different locations [25,26]. Additionally, the large-area and high-density design enhances the system’s robustness [27], ensuring that the remaining sensors continue to function effectively even if some units fail, thereby improving the overall reliability of the sensor system. Integrating flexible sensors with AIoT enhances the capability of the system to detect the condition of the surroundings by leveraging the excellent electrical properties of flexible sensors. Meanwhile, the unique mechanical properties of flexible sensors can enhance the flexibility and stretchability of the system. This broadens the potential applications of the AIoT system, driving the development of both flexible sensors and AIoT.

The key to integrating flexible sensors and AIoT lies in the incorporation of machine learning methods. The current methods of integrating flexible sensors with machine learning typically involve external computational assistance, utilizing the computing power of computers or cloud systems to achieve intelligent data analysis and recognition. AI-assisted flexible sensors have experienced widespread adoption [28–34]. However, computer-aided machine learning-based recognition methods [35–37] may encounter issues such as bandwidth limitations, energy inefficiency, and privacy concerns. External computation-assisted AI necessitates the transmission of data from local devices to either a computer or cloud to achieve intelligence. This data transmission process can be constrained by communication bandwidth, thereby impacting the efficiency of computations. Moreover, the energy-intensive nature of computers or the cloud computing centers leads to significant energy inefficiencies, contrasting with local processing units like microcontrollers (MCUs). Additionally, transmitting data to external entities exposes it to the risk of theft. On-chip computing can provide a viable solution to these challenges. On-chip computing decentralizes AI to local processing units, eliminating the necessity to transmit data to computers or the cloud. This reduces reliance on communication bandwidth, enhances computational efficiency, and mitigates privacy concerns. However, the extreme resource constraints of MCUs necessitate extensive optimization of the original neural network model to accommodate these limitations. Model compression techniques [38–41] can effectively reduce the parameter size and computational demands of a model, all while preserving a high recognition rate. Tiny machine learning (TinyML) for MCUs has garnered considerable interest from researchers worldwide [42–45]. Training memory consumption is excessively high for IoT devices with limited memory resources. In-sensor learning for neural networks is constrained by challenges such as difficult optimization of quantization maps and the inability to perform full backpropagation [45].

However, these methods only facilitate recognition and do not support in-sensor learning. Various factors, including changes in the sensor, environment, and user, can influence the recognition. The implementation of in-sensor adaptive learning can effectively address these challenges. Hyperdimensional (HD) computing [46,47], a novel machine learning approach, operates by encoding signals as hypervectors (HVs). With its inherent benefits of rapid learning, minimal recognition latency, and lightweight models, HD computing (HDC) proves to be a superior choice for in-sensor inference and learning compared to traditional machine learning methods [48]. HDC has been extensively utilized in diverse domains [49–53], encompassing speech recognition, language recognition, image recognition, and human gesture recognition. In-sensor learning via HDC is employed for real-time gesture recognition [49]. The system successfully achieves adaptive learning for different users and diverse environments, demonstrating the promising application prospects of HDC-based flexible sensing systems. However, the system utilizes a limited number of flexible electrode arrays for sensing, which can significantly impair recognition accuracy in the event of sensor damage. Simultaneously, the system employs field programmable gate arrays (FPGAs) for intelligent data processing, which can introduce challenges related to power consumption, integration, and cost.

This paper introduces a flexible, large-scale sensing array with low-power in-sensor intelligence to enhance the robustness against sensor damage. The array was created by bonding 2 composite layers (polyimide and copper) onto the top and bottom surfaces of the piezoresistive layer. Signal digitization from the large-scale resistor array is accomplished by utilizing row and column scanning. The in-sensor intelligent system used a compression HV encoder (CompHVE) to encode and process the large-scale pressure sensing array signals, thereby ensuring performances in recognition compared to separate HV encoder (SepHVE) or feature extraction HV encoder (FEHVE) when the dimension of HVs and the resolution of HV encoder increase. Low-power, low-latency, and accurate recognition and in-sensor learning were successfully implemented on an MCU with highly restrictive resource constraints (RAM: 20 kB; Flash: 64 kB). Finally, our system accomplished the precise categorization of 7 intrinsic states along with 5 additional newly identified states. In comparison to existing intelligent systems, the proposed flexible, large-scale, intelligent sensing array not only consumes fewer resources but also possesses in-sensor learning capability, ensuring outstanding robustness. With its exceptional flexibility, the system holds great potential for applications in smart cockpits, robotic haptics, human–machine interaction, and smart skins. Additionally, the capability for low-power in-sensor learning further enhances its ability to advance the development of the AIoT field.

## Results

### Design of flexible, intelligent sensing array

**Fig. 1.**
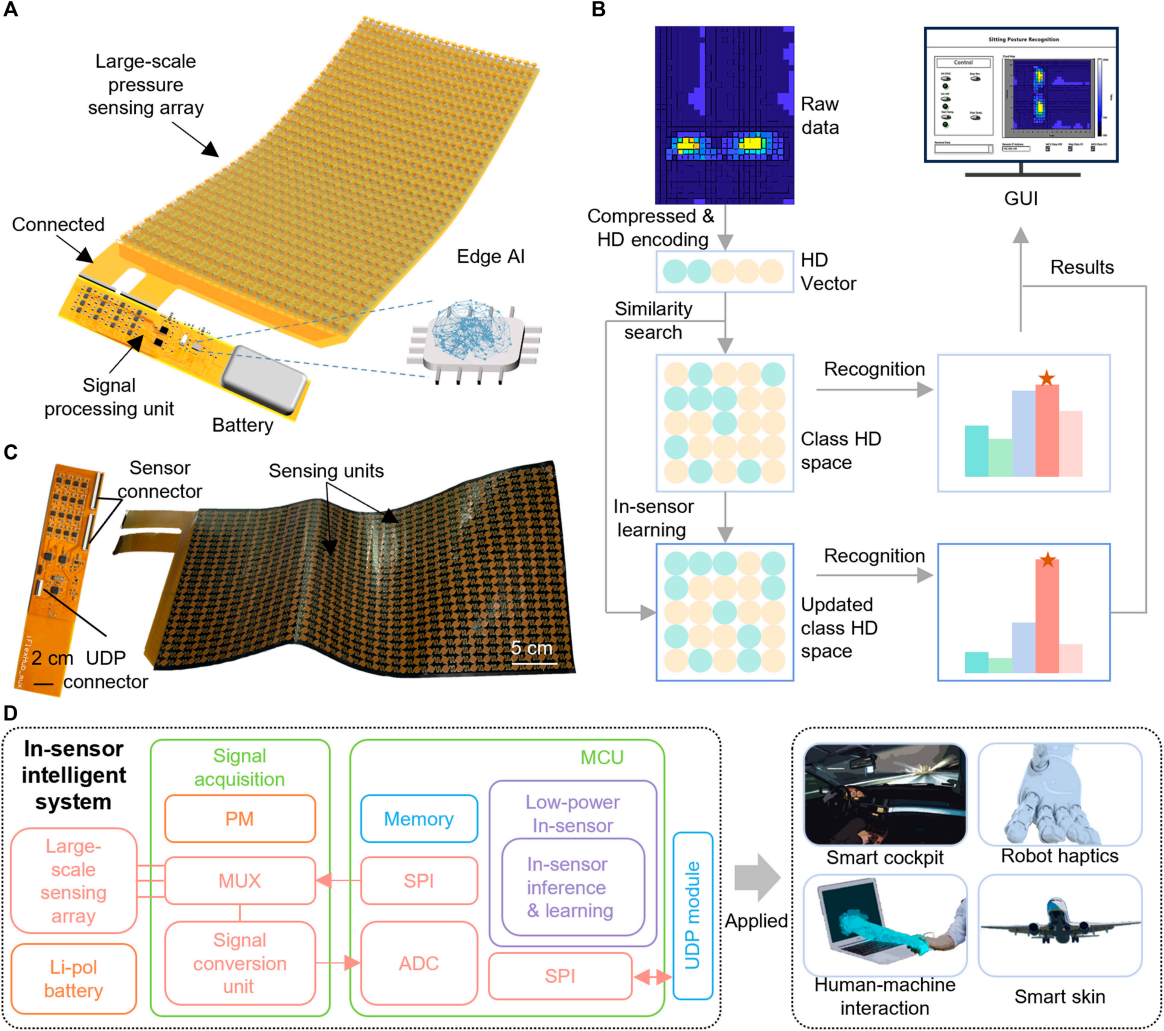
Schematic illustrations and images of a flexible, large-scale sensing array with low-power in-sensor intelligence. (A) Schematic illustration of the in-sensor intelligent system including a pressure sensing array, and a signal acquisition and processing module with in-sensor intelligent inference and learning capability. (B) Flowchart of in-sensor intelligent inference and learning method based on hyperdimensional (HD) computing. (C) Images of pressure sensing array and a signal acquisition and processing module. (D) Functional diagram of the signal measurement and in-sensor processing intelligent system and its potential applications.

Figure 1A illustrates a flexible intelligent sensing system enhanced by edge AI. The developed sensing system consists of 3 main components: (a) a large-scale flexible piezoresistive pressure sensing array (width, 240 mm; length, 500 mm) containing 800 sensing units for monitoring real-time changes in pressure, (b) a flexible signal acquisition and processing module for intelligent sensing, and (c) edge AI based on HDC for in-sensor inference and learning. Figure 1B presents the flow chart of the HDC algorithm for real-time inference and learning. The raw pressure data are compressed and converted into a query HVs using the HD encoder. Then, the normalized Hamming distance is calculated between these HVs with class HVs in the associative memory (AM) to recognize current states. The class HD space can be dynamically updated in real time to facilitate rapid learning. The capability of in-sensor learning allows the system to adapt to changes in the sensor, environment, and user. Both the raw pressure data and the recognition results can be displayed through the graphics user interface (GUI), which is also used to regulate the transmission of data and send labels for achieving supervised learning. The capture image of the GUI panel is shown in Fig. S1.

The flexible piezoresistive pressure sensing array is made up of a transparent polyurethane (PU) encapsulation layer, a piezoresistive layer (Velostat; thickness, ~100 μm), upper electrode layer [polyimide (PI)/Cu/PI; thickness, ~100 μm], and bottom electrode layer (PI/Cu/PI; thickness, ~100 μm), with the nonfunctional domain of electrode layer and the piezoresistive layer bonded by commercial adhesive (3M, Super 75), as shown in the top of Fig. S2. This sensing array can be integrated with seats (i.e., driver seats and office chairs) to gather pressure distribution. The flexibility and thickness of the prepared sensing array allow for pressure measurement without any sensation. The bottom of Fig. S2 shows the construction of the signal acquisition and processing module, which includes an MCU, power management IC (PMIC), and the signal conversion unit. The components are interconnected via a 2-layer flexible printed circuit board (fPCB) (PI/Cu/PI/Cu/PI; thickness, ~200 μm). Additionally, the built-in edge AI can quickly recognize various states according to the variations of pressure signal. The optical images of the flexible piezoresistive pressure sensing array and signal acquisition-processing module are presented in Fig. 1C. The acquisition-processing module is designed (width, 240 mm; length, 40 mm) to match the width of the sensing array. Two flexible printed circuit (FPC) connectors at the upper left of acquisition-processing module serve as the electrical interface between the sensing array and the acquisition module. The image depicting the connection between the pressure sensor and the signal acquisition module is shown in Fig. S3. The Lego-like signal processing module can be configured with different numbers of sensors for different application scenarios, as depicted in Fig. S4. The electrical interface between the acquisition module and a user datagram protocol (UDP) module for data transmission, as shown in Fig. S5, is also provided by an FPC connection at the lower part of acquisition-processing module. The acquisition module collects and converts the resistance values and then sends them via the UDP module.

Figure 1D illustrates the schematic diagram of the overall data flow and the potential applications of the flexible, intelligent sensing array. The entire system is powered by a lithium battery, with various voltage levels provided on demand by the PMIC. The upper and bottom electrodes of the sensing array are connected to two 32-channel multiplexers (MUX) through the 2 FPC connectors, respectively. To choose the single cell of sensing array, the MCU uses the serial peripheral interface (SPI) protocol to operate the multiplexers. The resistance variations of sensors are translated into voltage variations by the signal conversion processing unit (e.g., an inverse amplification circuit). Furthermore, the transformed voltage is digitalized through the integrated analog-to-digital converter (ADC) in the MCU, which has a measurement resolution of 12 bits. Subsequently, the digitalized signal is operated by the in-sensor processing algorithm stored in the memory of the MCU, which allows real-time in-sensor learning and inference. Finally, the MCU manages the UDP module via the SPI protocol and displays pressure data and recognition results in real time. The circuit schematic and PCB implementations of the flexible, intelligent sensing array are shown in Figs. S6 and S7, respectively. The flexibility and intelligence of the system enable a broad spectrum of applications in areas such as smart cockpits, robotic haptics, human–machine interaction, and smart skins.

### Characterization of piezoresistive sensor array

**Fig. 2.**
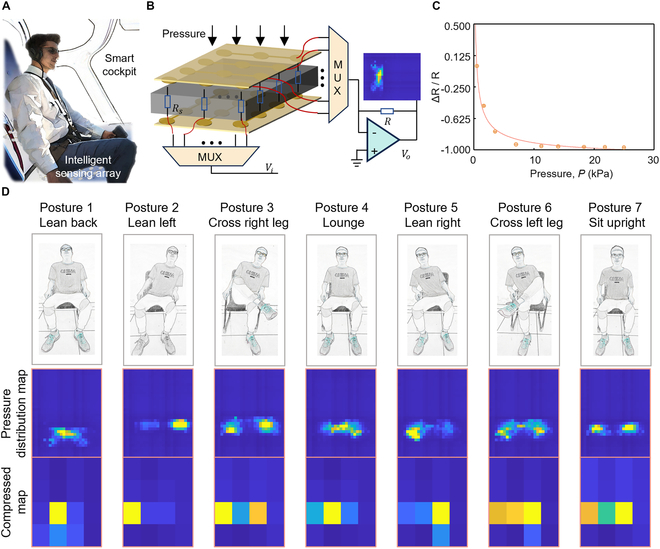
The configuration of the flexible piezoresistive sensing array and the pressure nephogram of different sitting postures. (A) Scene schematic of a smart cockpit equipped with intelligent sensing array. (B) Schematic illustration of sensor response, scanning measurement, and resistance conversion. (C) Response graph of resistance under different levels of external pressure. (D) Seven typical sitting postures (from left to right, lean backward, lean left, cross the right leg, lounge, lean right, cross the left leg, and sit straight), the pressure distribution map corresponding to various postures, and the compressed map.

Here, a smart cockpit serves as a typical application to exhibit the capability of the intelligent sensing array. Figure 2 shows the structural components and the performance of the flexible piezoresistive sensing array. Figure 2A illustrates key application scenarios for large-scale pressure sensing arrays in a smart seat-assisted cockpit. The sensing array signals are tested using relevant potential sitting postures. An ultrathin, flexible polymer conductive material [54] is utilized as the sensitive layer, known as Velostat material. The material mechanism is illustrated in Fig. S10. When the sensor is subjected to pressure, its internal particles compress. This compression enlarges the current path, leading to a reduction in the resistance. The relationship between resistance and pressure [54] is denoted by *R* = *f*(*σ*| *R*_0_), where *R* represents the sensor’s resistance, *R*_0_ represents the initial resistance of the sensor, and *σ* represents the applied stress. It can be observed that when *σ* changes, *R* changes accordingly.

Large-scale and high-density sensing arrays often require numerous connections. Using a row-column scanning method can effectively address this issue. The upper and bottom layers, with the design of row-column electrodes, reduce the number of connection wires, as shown in Fig. 2B. Through the MUX and inverse amplification circuit, the acquisition system scans each piezoresistive cell in sensing array to acquire the pressure signal. The resistance response of a piezoresistive sensing cell at various pressures is examined in Fig. 2C. The correlation between the change in the resistance value of sensing cell and the variation of the external pressure is articulated through a power function. The pressure range is set from 0 to 30 kPa after estimating the pressure on the seat. In the pressure range of 0 to 5 kPa, the resistance value decreases rapidly, with the sensor sensitivity being approximately 0.26 kPa^−1^. However, above 10 kPa, the resistance change stabilizes, and the sensor sensitivity reduces to around 0.002 kPa^−1^. The experimental results of sensing performance are shown in Fig. S11. Figure S11A presents the sensing detection limit of 147 Pa. Figure S11B shows the sensing pressure range. When the sensor is subjected to an external pressure ranging from 0 to 350 kPa, its resistance decreases gradually. Beyond 400 kPa, the sensor’s response becomes less pronounced. The operating range is set from 0 to 350 kPa to maintain optimal sensing performance. The measurement results of sensing response and recovery time are shown in Fig. S11C. Under a pressure of 10 kPa, the response time is approximately 0.1 s, and the recovery time is about 0.14 s. Figure S11D shows the sensing dynamic performance. During a pressure cycle with a maximum pressure of 10 kPa at a frequency of 0.1 Hz, the sensor’s resistance changes consistently with the pressure variations. The response of sensing array is demonstrated in Fig. 2D, corresponding to 7 possible sitting postures in daily work. From left to right, the sitting postures are leaning back, leaning left, crossing the right leg, lounging, leaning right, crossing the left leg, and sitting upright, respectively. The different sensing units exhibit excellent consistency, maintaining similar performance and response characteristics. This consistency ensures that the sensing array can provide highly reliable and accurate data during overall operation. The middle row of Fig. 2D shows the pressure distribution map (sampling rate of 10 Hz/channel) of the sensing array corresponding to various postures. Then, the pressure response map is calculated in real time for further processing by using an average compression method, as shown in the bottom row of Fig. 2D. Under various sitting postures, the compressed pressure responses of sensing arrays vary. The number of compressed data points (4 × 4 cells) is substantially smaller than before compression (32 × 32 cells), where the sensing response characteristics of various sitting postures remain with significant differences. More experiments are also conducted for other typical application scenarios. Figure S12 shows pressure response maps across various typical applications, including robotic haptics, human–machine interaction, and smart skin. Figure S12A and B demonstrates the sensing array’s response to pressure from a water cup and a laptop, illustrating its application in robotic tactile sensing. In human–machine interaction, a common application is palm pressing, as depicted in Fig. S12C. Figure S12D displays the response to an impact, representing a typical application in smart skin technology. Figure S12E displays the recognition results of the 4 scenarios. The intelligent sensing array can differentiate between various scenarios well.

### Real-time sitting posture recognition

**Fig. 3.**
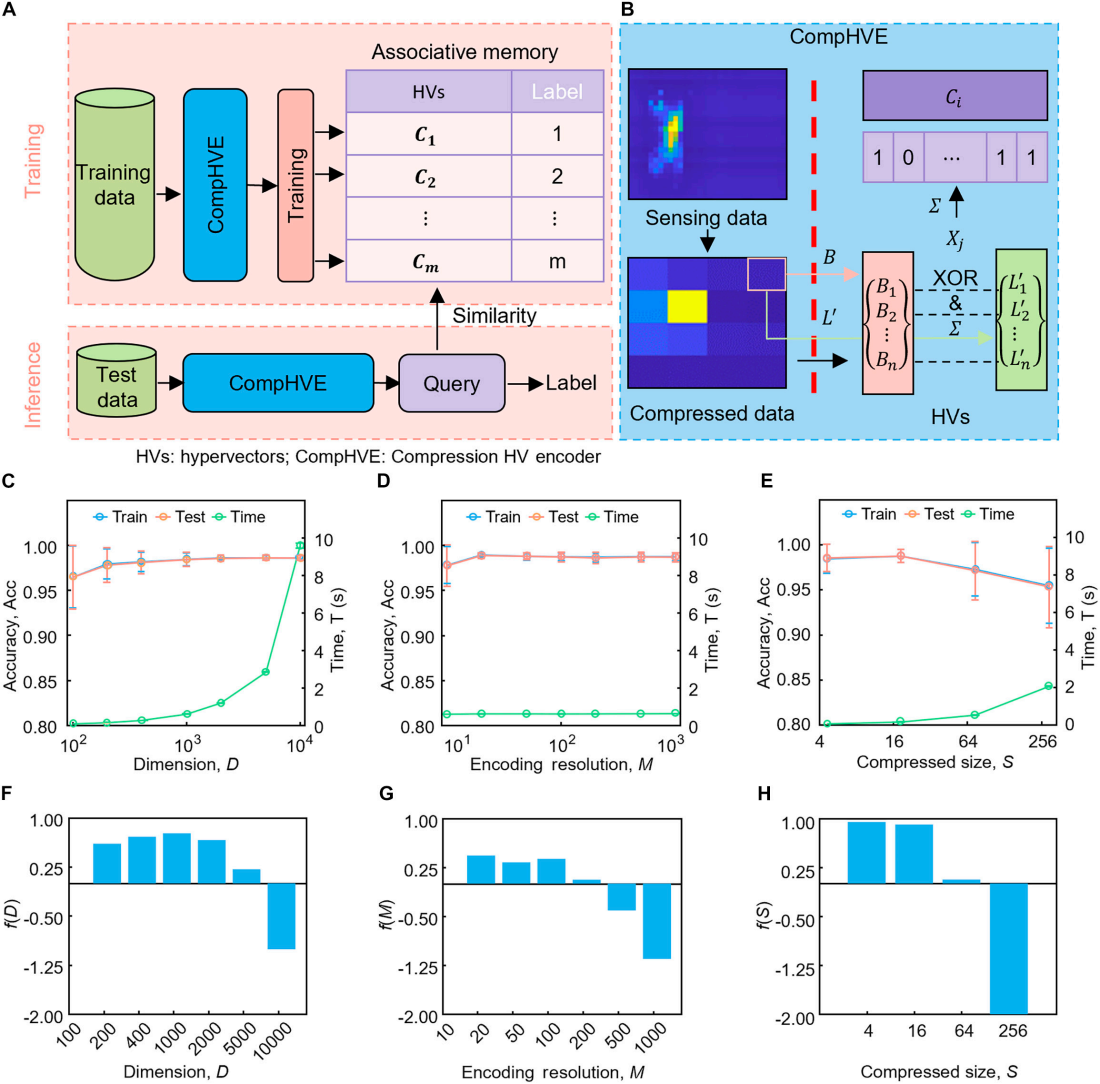
Implementation and improvements of HD computing. (A and B) Recognition principle of the compression hypervector (HV) encoder (CompHVE). (C to E) Performance improvements for CompHVE. Recognition accuracy and consumption time vary with dimension of HVs (C), resolution of HV encoder (D), and compressed image size (E). (F to H) Results of variation of the defined function *f* with *D*, *M*, and *S*. (F) Graph of the variation of function *f* with dimension. (G) Graph of the variation of function *f* with encoding resolution. (H) Graph of the variation of function *f* with compressed size.

The offline posture recognition is realized through HDC, the implementation flowchart of which is shown in Fig. 3A, including the training and inference section. The details of the compression HV encoder (CompHVE) are depicted in Fig. 3B. The gathered pressure data were paired with labels to represent various sitting postures and subsequently divided into training and test datasets based on a training ratio of 0.7. The top of Fig. 3A shows the training procedure of the HDC recognition algorithm, which involves classifying the sensing data of 7 different sitting postures to create the training dataset. The training dataset, consisting of 3,500 samples, was collected from a single subject in various sitting postures for offline training. As shown in Fig. S13, the raw data for the offline section illustrate the relationship between different samples on the *x* axis and various sensor channels on the *y* axis. The color in the image represents the values from the sensor channels. It is evident that there are significant differences in the raw data between different sitting postures, and even among samples of the same posture, there are some variations. A CompHVE is built for converting the sensing data of the flexible piezoresistive array, which is encoded using bipolar HVs [51] to create the HD space. The HVs of the same classification are added to obtain the corresponding categorized HV (***C****_i_*),

$$C_{i}=\sum_{j=1}^{n}H_{\mathrm{ij}}$$ (1)

where *i* is the *i*th label of the sitting posture, *n* is the number of training samples for the related classification, ***H****_ij_* represents the *j*th HVs of the *i*th posture, and Σ is the sum of multiple HVs. The arithmetic rules of multiple HVs refer to that defined in the previous work [46]. Eventually, different categorized HVs are stored in the AM.

The bottom of Fig. 3A presents the inference process of HDC for different sitting postures. The test data are encoded using CompHVE to obtain the query HVs, which are then utilized to compute the predicted label by determining their similarity with the trained model. CompHVE is used to obtain the query HV dataset, {***Q***_1_, ***Q***_2_, ⋯, ***Q****_n_*}, where *n* is the number of test samples. To determine the predicted categories, the Hamming distance is utilized to compute the similarity comparisons between all query HV and the different categorized HVs,

$$\mathrm{Ham}\left(Q_{j},C_{i}\right)=\frac{1}{D}\sum_{\alpha =1}^{D}1_{Q_{j}\left(\alpha \right)\neq C_{i}\left(\alpha \right)}$$ (2)

where ***C****_i_* is the *i*th categorized HV, ***Q****_j_* is the encoded HV query of the *j*th test sample, and *D* is the dimension of the HV. Due to the encoding method of bipolar splash code, the exclusive OR (XOR) operation can be expressed as multiplication [51]. Classification is achieved by finding the smallest Hamming distance. Eventually, the predicted categories are compared with the classification truth values, which are used to calculate the recognition accuracy and evaluate the system performance.

A CompHVE, shown in Fig. 3B, is built in the training and inference processes for encoding the raw sensing data of flexible piezoresistive array into HVs. First, an average compression method (average of 8 × 8 cells) is used to reduce the raw sensing array data (32 × 32 cells) to 4 × 4 cells. The robustness of the posture recognition is improved while the amount of data to be processed is decreased due to the compression method. As shown in Fig. S14, various sensor failure ratios (0.1, 0.2, 0.3, 0.4) are simulated to represent potential sensor failures in practical use. Figure S14A displays maps of the raw sensing, compressed, and feature data under different sensor failure ratios. These maps reveal noticeable changes in the data due to different sensor failure ratios. Figure S14B and C illustrates the impact of different sensor failure ratios on system training and testing recognition accuracy. The recognition accuracy of CompHVE remains above 90%. This indicates that the system maintains excellent robustness even in the presence of sensor failure. All pixel values for the entire compressed dataset are discretized to *m* different levels linearly. A list of HVs {***L***_1_, ***L***_2_, ⋯, ***L***_***m***_} is generated to represent each level. The minimum value of all pixel values is represented by the HV (***L***_1_), which is a *d*-dimensional HV generated randomly. Then, ***L****_i_* randomly flips *d*/*m* bits to generate ***L***_*i* + 1_, *i* ∈ [1, *m* − 1]. The last generated HV (***L****_m_*) represents the maximum value of all pixel values. The list of HVs generated by this process exhibits characteristics that ***L****_i_* and ***L***_*i* + 1_ are highly correlated, while ***L***_0_ and ***L****_m_* are nearly orthogonal, effectively reflecting the correlation within the data.

The updated HVs $\left\{{L_{1}}^{\;\prime }\;,{L_{2}}^{\;\prime }\;,\;\cdots ,{L_{n}}^{\;\prime }\right\}$ are generated by searching for the HV associated with the pixel values of the compressed data, where *n* is the compressed data size. The base HV set {***B***_1_, ***B***_2_, ⋯, ***B****_n_*} is generated randomly to represent the position of each pixel. The base HVs are nearly orthogonal [46] due to the randomness of HV generation. Each HV (***L***ʹ and ***B***) undergoes multiplication and addition [46] to obtain the encoded HV (***X***),

$$X=\sum_{j=1}^{n}B_{j}⊙L_{j}^{\prime }$$ (3)

where ***B****_j_* is the *j*th base HV of the pixel, $L_{j}^{\prime }$ is the *j*th updated HV, and *n* is the number of pixels. Finally, the addition is performed on ***X*** of the same posture to obtain the *i*th categorized HV (***C****_i_*).

The effects of the dimension of the HVs, the encoding resolution of the list of HVs, and the size of the compressed data are explored in terms of their impact on the accuracy and the time consumption of the algorithm based on CompHVE, as shown in Fig. 3C to E. In Fig. 3C, the accuracy and the robustness of the recognition for training dataset and test dataset increase as the number of dimension (*D*) increases, while the time consumption of this algorithm significantly increases by 2 orders of magnitude. Furthermore, an increase of HV dimension also results in a corresponding increase in memory requirements. Moreover, as shown in Fig. S15A, the recognition accuracy for the test and validation datasets are not significantly lower than those for the training dataset, indicating that overfitting did not occur during the offline training section. On the other hand, the L1 loss values for the test and validation datasets tend to be higher than those for the training dataset, which aligns with the recognition accuracy for the test and validation datasets being slightly lower than the training dataset. All 3 loss values remain relatively low, indicating that the model performs well in offline training (Fig. S15B). Additionally, as the training rates increase, the loss values decrease. Figure 3D illustrates the effect of encoding resolution (*M*) on the accuracy and time consumption of the algorithm. When *M* ≥ 40, the recognition accuracy and robustness remain unchanged, while encoding resolution does not have a significant effect on the time consumption (~1 s). The variation of recognition accuracy and time consumption versus the compressed data size is shown in Fig. 3E. The optimal size of compressed sensing arrays (*S*) for accuracy and robustness is *S* = 16, and the time consumption rises as the size of compressed data increases. To balance the effects of dimension (*D*), encoding resolution (*M*), and compressed size (*S*), a function  *f*(*D*, *M*, *S*) = *Norm*(*Acc*) − *Norm*(*T*) − *Norm*(*D* × *M* × *S*) is defined by normalizing the accuracy, time consumption, and occupied memory, respectively. The results of *f*(*D*, *M*, *S*) versus dimension (*D*), encoding resolution (*M*), and compressed size (*S*) are illustrated in Fig. 3F to H, respectively. Given available memory, *f* reaches its maximum at *D* = 1, 000, as shown in Fig. 3F. A plot of *f* versus resolution is shown in Fig. 3G, where it can be seen that *f* reaches its maximum value at *M* = 20. The effect of the compressed data size on the accuracy and consumed time is shown in Fig. 3H. *f* reaches its maximum value at *S* = 4. To guarantee the overall reliability of the system, the compressed size is chosen as 16 for real-time recognition.

### Comparison of different methods

**Fig. 4.**
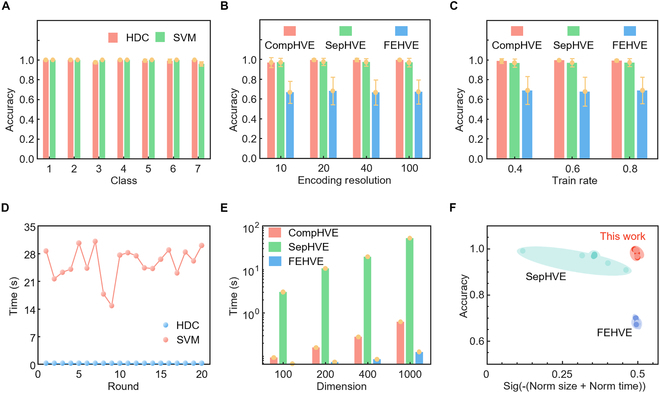
Comparison results of different methods. Accuracy (A) and inference time (D) comparisons of HDC and support vector machines (SVMs). Accuracy of 3 types of HDC including the compression HV encoder (CompHVE), the separate HV encoder (SepHVE), and the feature extraction HV encoder (FEHVE) with different encoding resolutions (B) and train rates (C). (E) Time comparisons of 3 types of HDC with different dimensions. (F) Ashby diagram of 3 types of HDC.

Figure 4 shows the comparison results of the recognition accuracy and time consumption of different methods, including machine learning algorithm [HDC and support vector machines (SVMs)] and in-sensor algorithm such as CompHVE, SepHVE, and FEHVE. Here, the results of comparison between HDC and the classical machine learning method (SVM) are shown in Fig. 4A and D. The recognition accuracy of HDC for the 7 different sitting postures was comparable to that of the SVM, both of which arrive at ~99% (Fig. 4A). But the HDC consumes less time (a few hundred milliseconds) to recognize the complete dataset than the SVM (around 25 s on average; Fig. 4D), which indicates that HDC is more suitable for in-sensor recognition systems with high real-time requirements.

In addition, Fig. 4B, C, E, and F compares the differences between CompHVE, SepHVE, and FEHVE. SepHVE encodes each unit of the sensing array with HVs, effectively preserving the original information of the sensing array. FEHVE, on the other hand, extracts spatial features from all sensing units and encodes the feature values [root mean square (RMS), median absolute deviation (MAD), mean, maximum, minimum, and kurtosis]. Among the combinations of these feature values, the combination of all features achieved the highest recognition accuracy. Therefore, this paper adopts the feature value combination as the method for FEHVE. Figure 4B shows that CompHVE exhibits higher robustness compared with the other 2 methods under different levels of encoding resolution for the sitting posture dataset. FEHVE has a lower accuracy (~60%), while CompHVE and SepHVE achieve high accuracy (~99%). For various train rates in Fig. 4C, CompHVE also demonstrates higher robustness with excellent accuracy. In terms of algorithmic time consumption (Fig. 4E), FEHVE takes less time than other 2 algorithms, and CompHVE consumes a similar amount of time, while the time consumption of SepHVE is more than 10 times as large. Figure S16 demonstrates that the recognition accuracy of CompHVE, SepHVE, and FEHVE all improve as the dimension increases. In these comparisons, invariant parameters have been optimized. Compared to FEHVE, CompHVE can significantly enhance the recognition rate (98.5%) with similar occupied memory and time consumption, as shown in Fig. 4F. Different encoders used the same parameters: *D* = 1,000, *M* = 40. Meanwhile, CompHVE can reduce occupied memory and time consumption while maintaining the recognition rate, as compared to the FEHVE approach.

### In-sensor recognition and learning

**Fig. 5.**
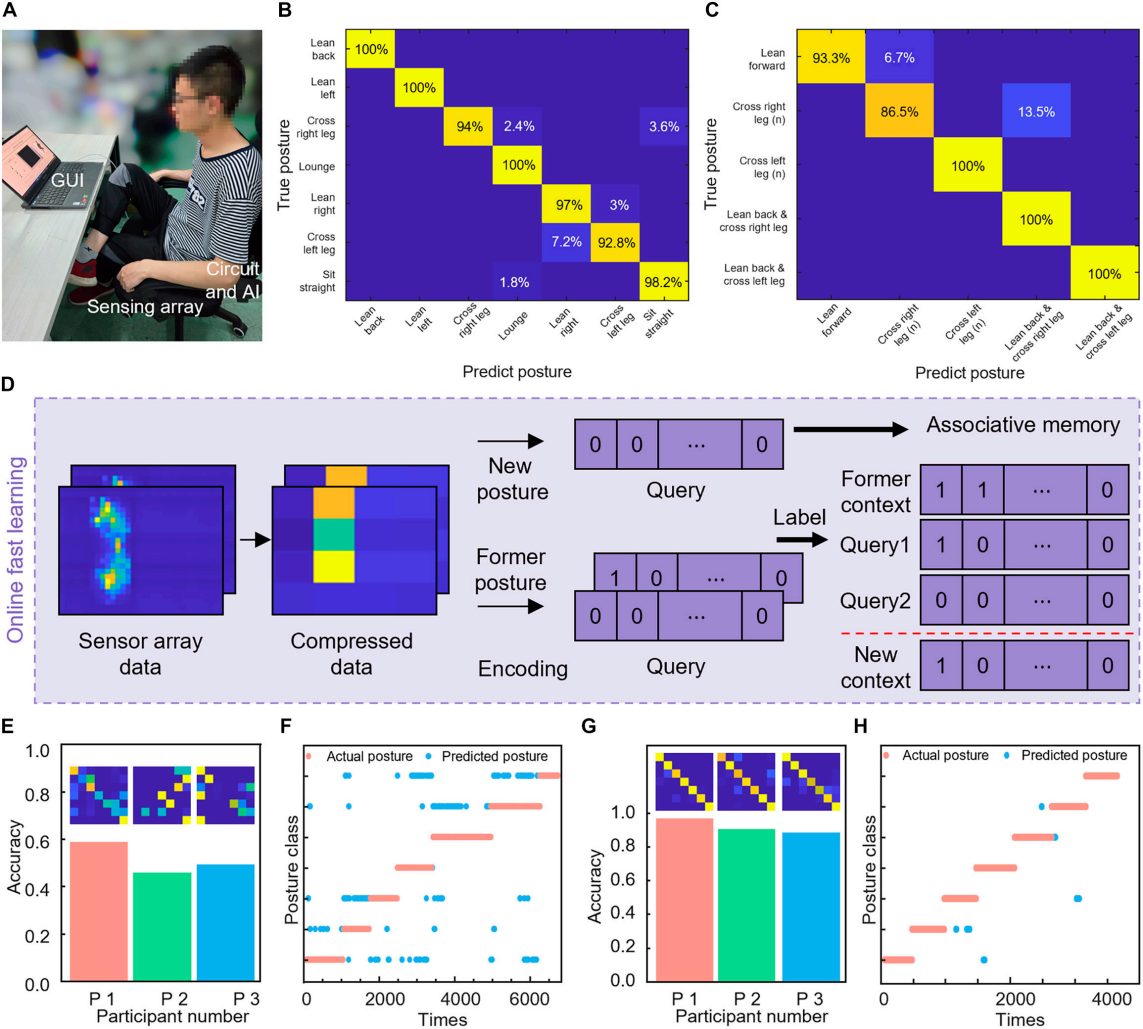
Implementation of in-sensor learning and the real-time classification results. (A) Scenario diagram for real-time experiments of pressure classification. (B) Classification confusion matrix of 7 former types of postures. (C) Classification confusion matrix of 5 new postures after in-sensor learning. (D) Principle of in-sensor learning. Average accuracy and classification confusion matrix of 3 participants before (E) and after (G) in-sensor learning. Real-time predicted postures and actual postures of participant no. 1 before (F) and after (H) in-sensor learning.

The CompHVE approach is utilized for the flexible, intelligent sensing array with real-time in-sensor inference and learning, the optimized parameters of which are selected as *D* = 1000, *M* = 20, and *S* = 16. The real-time sitting posture test scenarios are shown in Fig. 5A and Movie S1. The experiments obtain data from different sitting postures and classify the data to obtain the training dataset. The categorized HV set obtained from the training is stored in the memory of the MCU, including the corresponding minimum and maximum values, basis HVs, and HV lists. In the recognition experiments, the analog voltages from different sitting postures are converted into digital values in real time by the ADC unit, and the scanned signals are then reshaped and compressed in real time via the signal processing unit. The in-sensor inference module encodes the compressed signal into HVs in real time based on the stored corresponding minimum and maximum values and HV lists. The encoded HVs are multiplied by the stored base HVs, and the resulting HVs are summed to obtain the HV of the signal matrix in real time. Finally, the Hamming distance between the HV of the signal matrix and the different categorized HV set stored in the memory of the MCU is computed. The minimum value of the Hamming distance is searched for prediction. The in-sensor recognition system computes on chip and directly outputs the recognition results, thereby eliminating privacy concerns. Compared to computer-assisted recognition, the in-sensor recognition reduces the communication bandwidth requirement from 1,024 bytes to 1 byte. With the advancing era of IoT, the volume of sensor data accessed across networks is expected to increase, making the impact of reduced communication bandwidth requirements even more significant. In-sensor recognition significantly reduces communication bandwidth requirements compared to computer-assisted recognition, decreasing the rate from 122 kbps to 80 bps. As shown in Fig. S17A, the power consumption of the original MCU without in-sensor intelligence is 97 mW. When equipped with HDC, the power consumption increases to 106 mW, and with embedded neural networks (ENNs), it rises to 107 mW. In contrast, computer-assisted intelligence consumes 50 W, which is much higher than the power consumption of in-sensor intelligence. Figure S17B illustrates that HDC-based in-sensor intelligence has a delay of only 55.4 ms, whereas computer-assisted intelligence has a delay of approximately 373.4 ms. This indicates that, for this system, the HDC-based in-sensor intelligence offers outstanding delay performance.

Figure 5B presents the confusion matrix of in-sensor system for identifying 7 different sitting postures in real time. The results demonstrate that the system achieves a recognition rate of more than 90% for each of the 7 different sitting postures. The results presented in Fig. 5E are the accuracy and the confusion matrices of 3 new participants before in-sensor learning. The comparison of real-time predicted postures with actual postures of 3 participants before in-sensor learning is shown in Fig. 5F and Figs. S12A and C. The number of test results for different subjects before in-sensor learning was 3,600, 4,436, and 4,863, respectively. Due to the significant differences between the sitting postures of the participants and the training samples, the recognition accuracy of each participant is below 60% (i.e., 56.17%, 49.22%, and 54.45%) before in-sensor learning. Therefore, in-sensor learning is necessary to improve the recognition accuracy of postures for new participants. Figure 5D shows the schematic of the process of fast in-sensor learning. In-sensor learning allows the system to adapt to new sensor data while effectively preventing overfitting. The raw sensing data are processed through CompHVE to obtain the corresponding HV. The sum of an odd number of HV avoids the generation of randomness and simplifies operations. For the existing state in the AM, 2 different HVs (Query 1 and Query 2) of the same classification are summed with the HV of the corresponding classifications within the original AM to obtain a new HV, realizing the updating of the AM. For a new state that is not available in the AM, the HV encoded is stored directly in the AM to realize the in-sensor learning of the new state. Figure 5G presents the recognition accuracy and the confusion matrices of 3 new participants after the in-sensor learning described above. Figure 5H and Fig. S12B and D illustrate the comparison of real-time predicted postures with actual postures of 3 participants after in-sensor learning. The number of test results for different subjects after in-sensor learning was 6,819, 3,943, and 2,748, respectively. The recognition results show a significant improvement in recognition accuracy. The average value of the improved accuracy is 91%, all accuracy of the participants is above 85%, and the highest mean value accuracy reaches 96%. Compared to the results without in-sensor learning, the recognition accuracy improved from 56.17%, 49.22%, and 54.45% to 96.67%, 90.31%, and 88.16%, respectively.

In addition to adapting to different participants, the system facilitates the recognition of new poses through in-sensor HD learning. The system recognizes 5 new sitting postures in real time after fast in-sensor learning of the postures. The confusion matrix of the new sitting posture test results is shown in Fig. 5C. As shown in Fig. S11, there are 5 new sitting postures from left to right: leaning forward, new crossing left leg, new crossing right leg, leaning back and crossing left leg, and leaning back and crossing right leg. The recognition accuracy for new sitting postures all reached >85%, with some postures arriving at 100% as well. These results show that in-sensor HD learning can significantly ensure the recognition rate of new sitting postures with excellent adaptability. Because of the reduced latency, lower power consumption, and enhanced privacy, in-sensor learning is also applied in scenarios such as gesture recognition [49], drones [55], and computer vision [45]. In-sensor learning represents a marked leap forward in the integration of sensing and processing, paving the way for more intelligent and responsive systems across a wide array of applications.

## Discussion

This paper presents the design and implementation of a flexible, intelligent sensing array that integrates in-sensor inference and learning with HDC. To ensure robust monitoring of pressure distribution, a flexible piezoresistive sensor array consisting of 2 layers of fPCB and a pressure-sensitive layer is developed. Several modules are proposed to convert and analyze the sensor signals. The operational amplifier-based resistance conversion module is designed to convert the sensor response into voltage changes, and the row and column scanning module is used for converting large-scale resistor arrays. An in-sensor analysis module is proposed for real-time monitoring, inference, and learning. A power management module has been designed to provide the necessary voltage levels for different modules. Furthermore, a communication module is implemented to transmit both the original data and real-time in-sensor inference results. The compression hypervector encoder (CompHVE) technique is developed to achieve robust encoding and recognition. Despite highly restrictive resource constraints (RAM: 20 kB; Flash: 64 kB), low-latency recognition and in-sensor learning are successfully implemented on an MCU. Our results demonstrate the recognition of 7 states and the local learning of 5 new states using the proposed flexible, in-sensor intelligent system. The system can achieve a recognition accuracy of more than 85% for various states and generally exceeds 90%. The in-sensor recognition reduces the communication bandwidth requirement from 1,024 bytes to 1 byte, and reduces energy consumption from ~50 W to ~100 mW. The system holds great potential for applications in smart cockpits, robotic haptics, human–machine interaction, and smart skins. Alongside the innovative developments presented in this study, there are 2 key limitations. First, the current implementation of in-sensor intelligence relies on supervised learning. Supervised learning requires a computer to send labels to aid the learning process. This will reduce the system’s integration level. Second, this study primarily addresses the digital implementation of the sensing array with in-sensor intelligence. The reliance on digital computing resources can limit the real-time capabilities and adaptability of the sensing systems. Integrating analog devices and unsupervised learning could offer advantages, such as eliminating the need for an ADC, reducing power consumption, and expanding the applicability. Future work will focus on advancing analog implementations and exploring unsupervised learning approaches for the flexible sensing array.

## Materials and Methods

### Fabrication of the pressure sensing array and the signal acquisition and processing module

The top electrode layer and the bottom electrode layer of the sensor array are made of fPCB (50 μm, PI; 35 μm, Cu; 25 μm, PI; 35 μm, Cu; 50 μm, PI). The top electrode layer contains 25 rows of nodes, each row containing 32 pads. The bottom electrode layer has 32 rows of nodes, each containing 25 pads. The thermal release tape is patterned and adhered to the top and bottom electrode layer, and then 3M adhesive is sprayed. The tape loses adhesion when heated to 125 °C and is removed. The top and bottom electrode layer is pasted to the sensitivity layer, and PU film is pasted to the top electrode layer for waterproofing (Fig. S8). The fPCB for signal acquisition and processing is manufactured with the same parameters (50 μm, PI; 35 μm, Cu; 25 μm, PI; 35 μm, Cu; 50 μm, PI). The components on top of the fPCB include 3 DC–DC chips (Texas Instruments, TPS63001; Texas Instruments, TPS7A3725; and Texas Instruments, TPS63710) for power management, an MCU (STMicroelectronics, STM32F103C8T6) for analog-to-digital conversion, sending data to the host computer and on-chip real-time recognition and learning, 2 multiplexers (Analog Devices, ADG731) for row and column scanning, and 16 operational amplifiers (Texas Instruments, NE5532) for converting the resistive signals of the sensors to voltage signals, respectively.

### Experimental test of piezoresistive sensor array characterization

The measuring equipment of the sensor is shown in Fig. S9, where a manometer (ZHIQU, ZQ-21B-1) is used for pressure application and measurement. The size of the force application unit of the manometer is 10 mm × 10 mm. A multimeter (Keysight, 34465A) is used to measure the change of the sensor resistance value. When the force application unit of the manometer moves downward, it applies pressure to the sensor, displaying a pressure indication. Following the pressure application, the resistance undergoes real-time measurement using a multimeter.

### In-sensor recognition and learning on the MCU

The flowchart for in-sensor recognition and learning on the MCU is shown in Fig. S10. After initialization of the system, it will determine whether it receives the instruction to learn. When it does not receive the instruction to learn, the system will enter the recognition state. The system in the recognition state will continuously scan the sensing data. When the scan is finished, it will read the scanned data and then calculate the Hamming distance between the HDC obtained by on-chip CompHVE and the stored categorized HVs. Finally, recognition results are obtained by minimum value search. When a learning command is received, the system enters the learning state. The system in the learning state first gets the supervised labels, then compresses and encodes the scanned data, and finally adds the encoded HDC to the AM. The in-sensor recognition process primarily involves the MCU compressing the sampled data from 2,048 bytes to 32 bytes through a compression method. These compressed data are then quantized and encoded by an HV encoder. The HV encoder and the corresponding base HVs are pre-generated by a computer and stored as an array in the memory of the MCU, where they remain unchanged. Each bit is extracted and processed through shifting operations for addition and XOR. The encoded HVs are then used to calculate the Hamming distance with the HVs stored in a dynamically updated array, enabling real-time recognition of sitting posture. The dynamically updated array is referred to as AM. In-sensor learning is achieved by dynamically updating the HVs in the AM.

## Data Availability

All data are available in the manuscript or the Supplementary Materials or from the author.

## References

[1] Zhang J, Tao DC. Empowering things with intelligence: A survey of the progress, challenges, and opportunities in artificial intelligence of things. IEEE Internet Things. 2021;8(10):7789–7817.

[2] Wang YH, Yin L, Bai YZ, Liu SY, Wang L, Zhou Y, Hou C, Yang ZY, Wu H, Ma JJ, et al. Electrically compensated, tattoo-like electrodes for epidermal electrophysiology at scale. Sci Adv. 2020;6(43):eabd0996. 33097545 10.1126/sciadv.abd0996 PMC7608837

[3] Zhang F, Li SP, Shen ZM, Cheng X, Xue ZG, Zhang H, Song HL, Bai K, Yan DJ, Wang HL, et al. Rapidly deployable and morphable 3D mesostructures with applications in multimodal biomedical devices. Proc Natl Acad Sci USA. 2021;118(11): Article e2026414118. 33836614 10.1073/pnas.2026414118 PMC7980465

[4] Song HL, Luo GQ, Ji ZY, Bo RH, Xue ZG, Yan DJ, Zhang F, Bai K, Liu JX, Cheng X, et al. Highly-integrated, miniaturized, stretchable electronic systems based on stacked multilayer network materials. Sci Adv. 2022;8(11):eabm3785. 35294232 10.1126/sciadv.abm3785 PMC8926335

[5] Jin TQ, Cheng X, Xu SW, Lai YC, Zhang YH. Deep learning aided inverse design of the buckling-guided assembly for 3d frame structures. J Mech Phys Solids. 2023;179: Article 105398.

[6] Yang SY, Sharma P. A tutorial on the stability and bifurcation analysis of the electromechanical behaviour of soft materials. Appl Mech Rev. 2023;75(4): Article 044801.

[7] Yao XC, Li M, He SC, Jing LQ, Li CM, Tao J, Hui XN, Gao F, Song JZ, Chen HS, et al. Kirigami-triggered spoof plasmonic interconnects for radiofrequency elastronics. Research. 2024;7:0367. 38694204 10.34133/research.0367 PMC11062506

[8] Yu HY, Bian J, Chen FR, Li K, Huang YA. Laser-guided, self-confined graphitization for high-conductivity embedded electronics. Research. 2024;7:0305. 38628354 10.34133/research.0305 PMC11020139

[9] Ma YJ, Zhang YC, Cai SS, Han ZY, Liu X, Wang FL, Cao Y, Wang ZH, Li HF, Chen YH, et al. Flexible hybrid electronics for digital healthcare. Adv Mater. 2020;32(15):1902062. 10.1002/adma.201902062 31243834

[10] Zhuang MQ, Yin L, Wang YH, Bai YZ, Zhan J, Hou C, Yin LT, Xu ZY, Tan XH, Huang YA. Highly robust and wearable facial expression recognition via deep-learning-assisted, soft epidermal electronics. Research. 2021;2021:9759601. 34368767 10.34133/2021/9759601 PMC8302843

[11] Bai YZ, Yin LT, Hou C, Zhou YL, Zhang F, Xu ZY, Li K, Huang YA. Response regulation for epidermal fabric strain sensors via mechanical strategy. Adv Funct Mater. 2023;33(31):2214119.

[12] Matthew G, Ira S, Bruno R, Nathan Z, Sara K, Hyeonseok K, Woon-Hong Y. Wireless batteryless soft sensors for ambulatory cardiovascular health monitoring. Soft Sci. 2023;3(3):23.

[13] Dahiya R, Yogeswaran N, Liu FY, Manjakkal L, Burdet E, Hayward V, Jorntell H. Large-area soft e-skin: The challenges beyond sensor designs. Proc IEEE. 2019;107(10):2016–2033.

[14] Shi JL, Dai Y, Cheng Y, Xie S, Li G, Liu Y, Wang JX, Zhang RR, Bai NN, Cai MK, et al. Embedment of sensing elements for robust, highly sensitive, and cross-talk-free iontronic skins for robotics applications. Sci Adv. 2023;9(9):eadf8831. 36867698 10.1126/sciadv.adf8831 PMC9984179

[15] Xiong WN, Zhang F, Qu SY, Yin LT, Li K, Huang YA. Marangoni-driven deterministic formation of softer, hollow microstructures for sensitivity-enhanced tactile system. Nat Commun. 2024;15(1):5596. 38961075 10.1038/s41467-024-49864-z PMC11222500

[16] Tian SR, Yl W, Deng HT, Wang Y, Zhang XS. Flexible pressure and temperature sensors towards e-skin: Material, mechanism, structure and fabrication. Soft Sci. 2023;3(3):30.

[17] Tang X, Shen H, Zhao SY, Li N, Liu J. Flexible brain-computer interfaces. Nat Electron. 2023;6(2):109–118.

[18] Liu ZJ, Tian B, Jiang ZD, Li SM, Lei JM, Zhang ZK, Liu JJ, Shi P, Lin QJ. Flexible temperature sensor with high sensitivity ranging from liquid nitrogen temperature to 1200 °c. Int J Extrem Manuf. 2023;5(1): Article 015601.

[19] Pritish N, Samira P, Sanghoon L. Prospects of soft biopotential interfaces for wearable human-machine interactive devices and applications. Soft Sci. 2023;3(3):24.

[20] Xiong WN, Zhu C, Guo DL, Hou C, Yang ZX, Xu ZY, Qiu L, Yang H, Li K, Huang YA. Bio-inspired, intelligent flexible sensing skin for multifunctional flying perception. Nano Energy. 2021;90: Article 106550.

[21] Zhu C, Xu ZY, Hou C, Lv XD, Jiang S, Ye D, Huang YA. Flexible, monolithic piezoelectric sensors for large-area structural impact monitoring via music-assisted machine learning. Struct Health Monit. 2024;23(1):121–136.

[22] Huang YA, Zhu C, Xiong WN, Wang Y, Jiang YG, Qiu L, Guo DL, Hou C, Jiang S, Yang ZX, et al. Flexible smart sensing skin for “fly-by-feel” morphing aircraft. Sci China Technol Sci. 2021;65:1–29.

[23] Nela L, Tang JS, Cao Q, Tulevski G, Han SJ. Large-area high-performance flexible pressure sensor with carbon nanotube active matrix for electronic skin. Nano Lett. 2018;18(3):2054–2059. 29442518 10.1021/acs.nanolett.8b00063

[24] Wu XD, Khan Y, Ting J, Zhu J, Ono S, Zhang XX, Du SX, Evans JW, Lu CH, Arias AC. Large-area fabrication of high-performance flexible and wearable pressure sensors. Adv Electron Mater. 2020;6(2):1901310.

[25] Sundaram S, Kellnhofer P, Li YZ, Zhu JY, Torralba A, Matusik W. Learning the signatures of the human grasp using a scalable tactile glove. Nature. 2019;569(7758):698–702. 31142856 10.1038/s41586-019-1234-z

[26] Luo YY, Li YZ, Sharma P, Shou W, Wu K, Foshey M, Li BC, Palacios T, Torralba A, Matusik W. Learning human-environment interactions using conformal tactile textiles. Nat Electron. 2021;4(3):193–201.

[27] Shao LL, Lei T, Huang TC, Bao ZN and Cheng KT. Robust design of large area flexible electronics via compressed sensing. Paper presented at: Proceedings of the 57th ACM/EDAC/IEEE Design Automation Conference (DAC); 2020; San Francisco: CA, USA.

[28] Jin XF, Liu CH, Xu TL, Su L, Zhang XJ. Artificial intelligence biosensors: Challenges and prospects. Biosens Bioelectron. 2020;165: Article 112412. 32729531 10.1016/j.bios.2020.112412

[29] Wang M, Wang T, Luo YF, He K, Pan L, Li Z, Cui ZQ, Liu ZH, Tu JQ, Chen XD. Fusing stretchable sensing technology with machine learning for human-machine interfaces. Adv Funct Mater. 2021;31(39):2008807.

[30] Liu MW, Zhang YJ, Tao TH. Recent progress in bio-integrated intelligent sensing system. Adv Intell Syst. 2022;4(6):2100280.

[31] Niu HS, Yin FF, Kim ES, Wang WX, Yoon D, Wang C, Liang JE, Li Y, Kim NY. Advances in flexible sensors for intelligent perception system enhanced by artificial intelligence. InfoMat. 2023;5(5): Article e12412.

[32] Sun TM, Feng B, Huo JP, Xiao Y, Wang WA, Peng J, Li ZH, Du CJ, Wang WX, Zou GS, et al. Artificial intelligence meets flexible sensors: Emerging smart flexible sensing systems driven by machine learning and artificial synapses. Nano-Micro Lett. 2024;16(1):14. 10.1007/s40820-023-01235-x PMC10643743 37955844

[33] Xu CH, Solomon SA, Gao W. Artificial intelligence-powered electronic skin. Nat Mach Intell. 2023;5(11):1344–1355. 38370145 10.1038/s42256-023-00760-z PMC10868719

[34] Vu CC. Embedded-machine learning and soft, flexible sensors for wearable devices - viewing from an ai engineer. Mater Today Phys. 2024;42: Article 101376.

[35] Shi WS, Cao J, Zhang Q, Li YHZ, Xu LY. Edge computing: Vision and challenges. IEEE Internet Things. 2016;3(5):637–646.

[36] Banitalebi-Dehkordi A, Vedula N, Pei J, Xia F, Wang LJ and Zhang Y. Auto-split: A general framework of collaborative edge-cloud AI. Paper presented at: Proceedings of the 27th ACM SIGKDD Conference on Knowledge Discovery & Data Mining; 2021; New York, NY, USA.

[37] Gill SS, Tuli S, Xu MX, Singh I, Singh KV, Lindsay D, Tuli S, Smirnova D, Singh M, Jain U, et al. Transformative effects of IoT, blockchain and artificial intelligence on cloud computing: Evolution, vision, trends and open challenges. Internet Things. 2019;8: Article 100118.

[38] Han S, Pool J, Tran J and Dally WJ. Learning both weights and connections for efficient neural networks. arXiv. 2015. 10.48550/arXiv.1506.02626.

[39] Gupta S, Agrawal A, Gopalakrishnan K and Narayanan P. Deep learning with limited numerical precision. Paper presented at: International Conference on Machine Learning; 2015; Lille, France.

[40] Han S, Mao HZ and Dally WJ. Deep compression: Compressing deep neural networks with pruning, trained quantization and Huffman coding. arXiv. 2015. 10.48550/arXiv.1510.00149.

[41] Yao SC, Zhao YR, Zhang A, Su L and Abdelzaher T. Deepiot: Compressing deep neural network structures for sensing systems with a compressor-critic framework. Paper presented at: Proceedings of the 15th Acm Conference on Embedded Networked Sensor Systems (Sensys’17); 2017; Hangzhou, China.

[42] Saha SS, Sandha SS, Srivastava M. Machine learning for microcontroller-class hardware: A review. IEEE Sensors J. 2022;22(22):21362–21390. 10.1109/jsen.2022.3210773 PMC9683383 36439060

[43] Lin J, Chen W-M, Lin YJ, Cohn J, Gan C, Han S. MCUNet: Tiny deep learning on IoT devices. arXiv. 2020. 10.48550/arXiv.2007.10319.

[44] Lin J, Chen W-M, Cai H, Gan C, Han S. MCUNetV2: Memory-efficient patch-based inference for tiny deep learning. arXiv. 2021. 10.48550/arXiv.2110.15352.

[45] Lin J, Zhu LG, Chen W-M, Wang W-C, Gan C, Han S. On-device training under 256KB memory. arXiv. 2022. 10.48550/arXiv.2206.15472.

[46] Kanerva P. Hyperdimensional computing: An introduction to computing in distributed representation with high-dimensional random vectors. Cogn Comput. 2009;1(2):139–159.

[47] Kanerva P. Computing with high-dimensional vectors. IEEE Des Test. 2019;36(3):7–14.

[48] Ge LL, Parhi KK. Classification using hyperdimensional computing: A review. IEEE Circ Syst Mag. 2020;20(2):30–47.

[49] Moin A, Zhou A, Rahimi A, Menon A, Benatti S, Alexandrov G, Tamakloe S, Ting J, Yamamoto N, Khan Y, et al. A wearable biosensing system with in-sensor adaptive machine learning for hand gesture recognition. Nat Electron. 2020;4:54–63.

[50] Kleyko D, Davies M, Frady EP, Kanerva P, Kent SJ, Olshausen BA, Osipov E, Rabaey JM, Rachkovskij DA, Rahimi A, et al. Vector symbolic architectures as a computing framework for emerging hardware. Proc IEEE. 2022;110(10):1538–1571. PMC10588678 37868615

[51] Kleyko D, Rachkovskij D, Osipov E, Rahimi A. A survey on hyperdimensional computing aka vector symbolic architectures, part II: Applications, cognitive models, and challenges. ACM Comput Surv. 2023;55(9):1–52.

[52] Kleyko D, Rachkovskij DA, Osipov E, Rahimi A. A survey on hyperdimensional computing aka vector symbolic architectures, part I: Models and data transformations. ACM Comput Surv. 2023;55(6):1–40.

[53] Schlegel K, Neubert P, Protzel P. A comparison of vector symbolic architectures. Artif Intell Rev. 2021;55(6):4523–4555.

[54] Yuan LQ, Qu HW, Li J. Velostat sensor array for object recognition. IEEE Sensors J. 2022;22(2):1692–1704.

[55] Mitrokhin A, Sutor P, Fermuller C, Aloimonos Y. Learning sensorimotor control with neuromorphic sensors: Toward hyperdimensional active perception. Sci Robot. 2019;4(30):eaaw6736. 33137724 10.1126/scirobotics.aaw6736

