# Supplementary material for: A Flexible, Large-Scale Sensing Array with Low-Power In-Sensor Intelligence
Source: Research (Wash D C). 2024 Nov 13;7:0497. doi: 10.34133/research.0497 (PMC11558032; doi:10.34133/research.0497)
Supplement: Supplementary 1 — Figs. S1 to S20 Movie S1 [file research.0497.f1.zip › Re - SI - Sensing Array with In-sensor Intelligence.pdf]

# Supplementary Information

# A flexible, large-scale sensing array with low-power in-sensor intelligence

Zhangyu Xu[1,2†], Fan Zhang[1,2†], Chao Hou[1,2], Erxuan Xie[1,2], Liting Yin[1,2], Hanqing Liu[1,2], Mengfei Yin[1,2], Lang Yin[1,2], Xuejun Liu[1,2], YongAn Huang[1,2*]

[1] State Key Laboratory of Intelligent Manufacturing Equipment and Technology, Huazhong University of Science and Technology, Wuhan, 430074, China

[2] Flexible Electronics Research Center, Huazhong University of Science and Technology, Wuhan, 430074, China

[†] The authors contribute equally.

[*] The corresponding author.

Email: yahuang@hust.edu.cn (Y.H.)

## Supplementary information

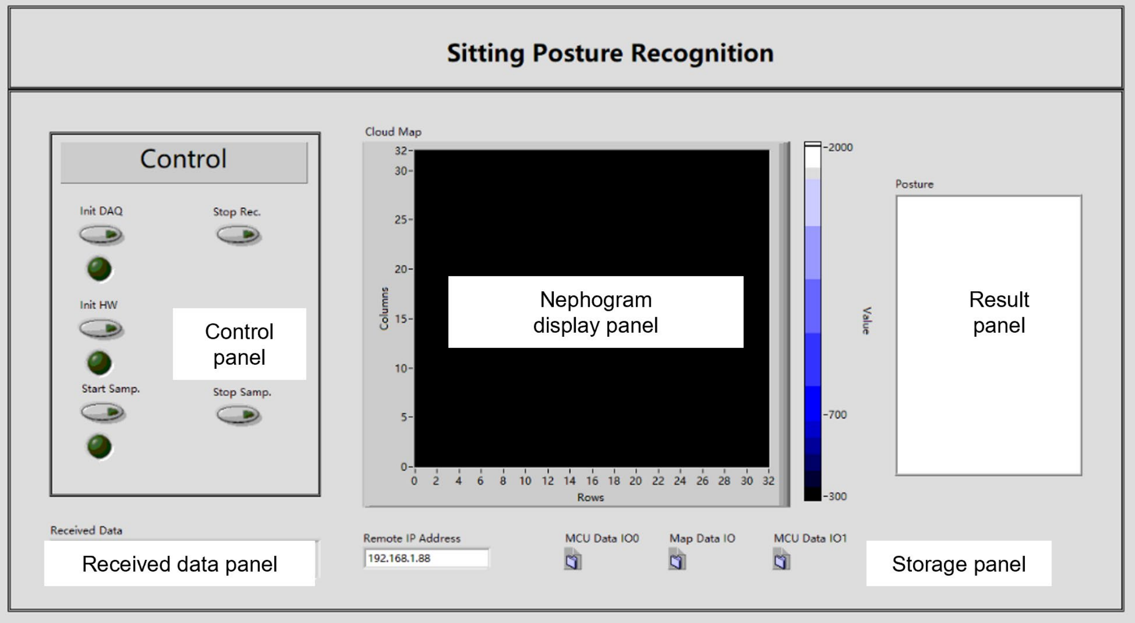


**Fig. S1.** The graphics user interface (GUI) for data reception, cloud map display, data storage and control.

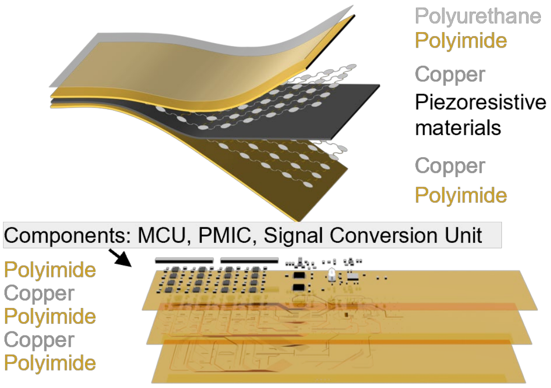


**Fig. S2.** Exploded view illustration of flexible pressure sensing array and flexible printed circuit board (fPCB) used for data acquisition and intelligent processing.

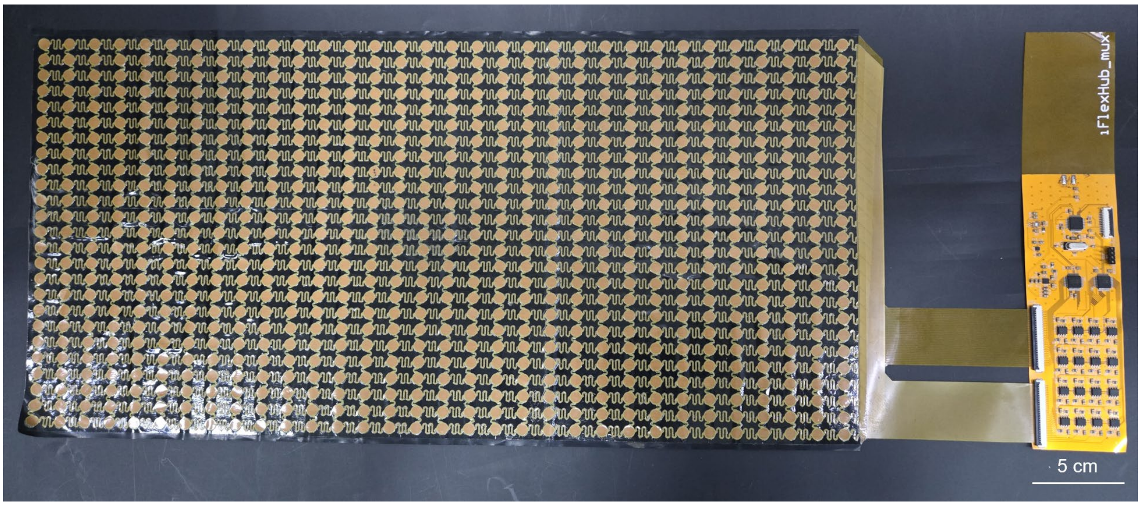


**Fig. S3.** Image depicting the connection between the pressure sensor and the signal acquisition module.

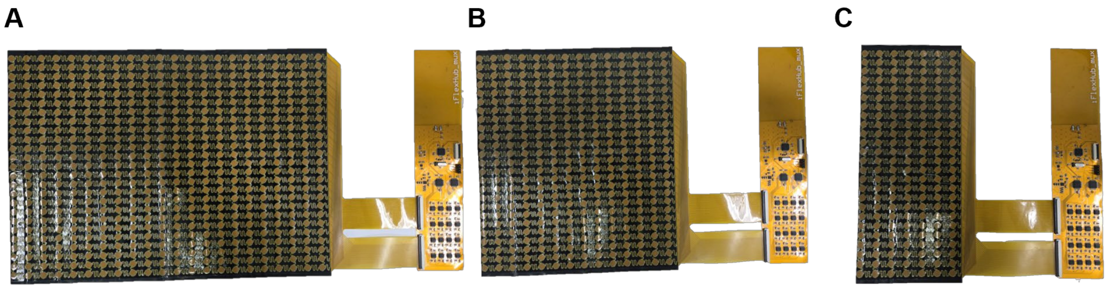


**Fig. S4.** Image of a Lego-like intelligent signal processing module configured with different numbers of sensors: five hundred and fifty (A), three hundred and fifty (B), and one hundred and seventy-five (C).

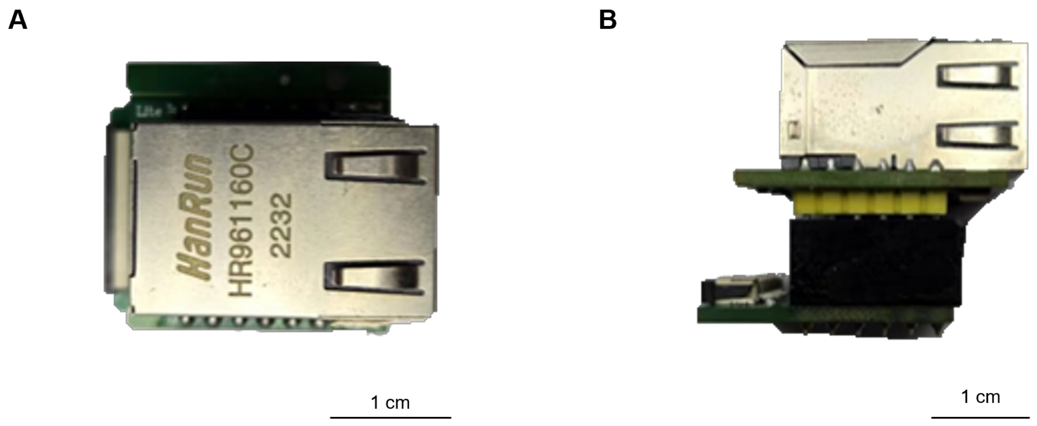


**Fig. S5.** Top view (A) and Front view (B) of the user datagram protocol (UDP) module.

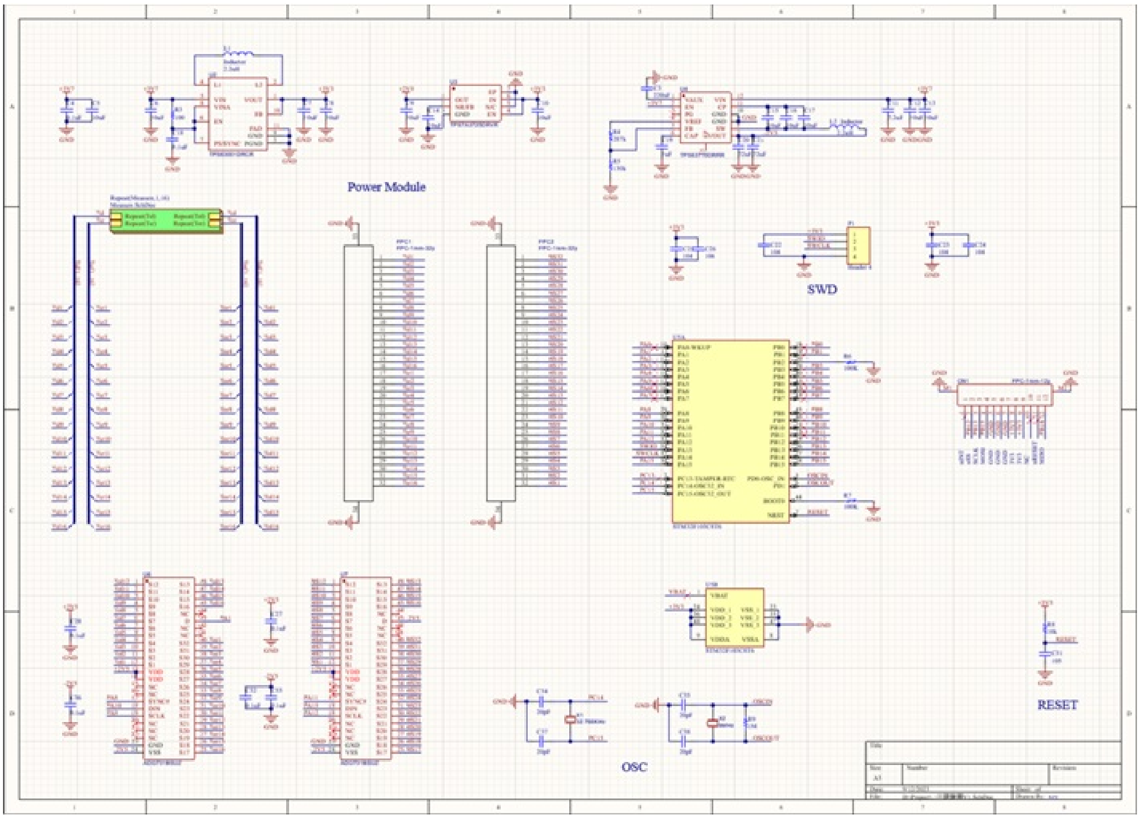


**Fig. S6.** The schematic implementations for power management, data acquisition, on-chip data analysis and data transmission.

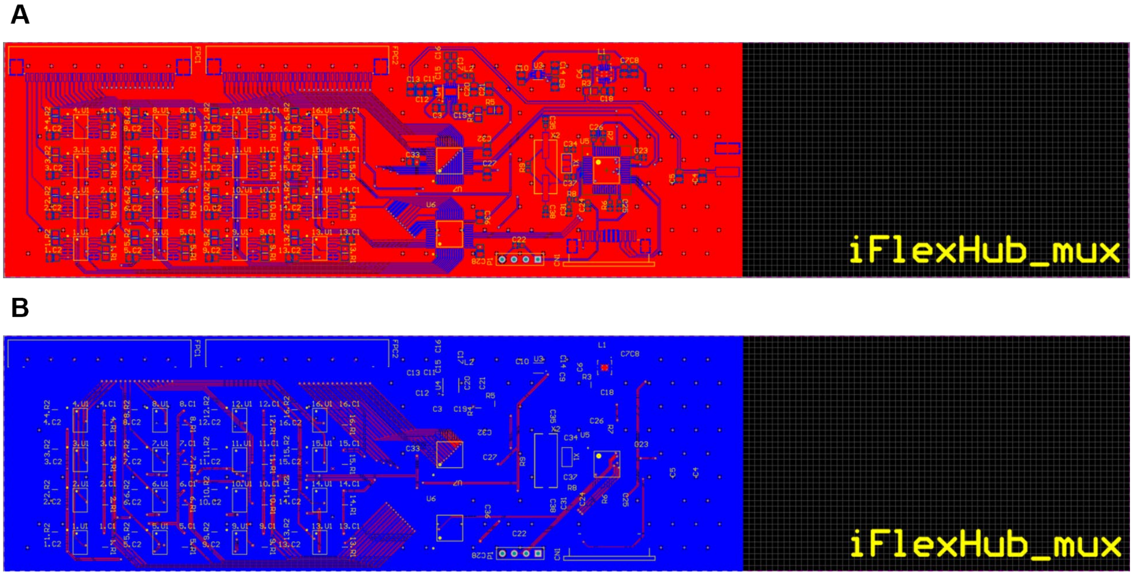


**Fig. S7.** The printed circuit board (PCB) implementation of the schematic in Fig. S3. Top layer (A) and bottom layer (B) of the designed PCB diagram.

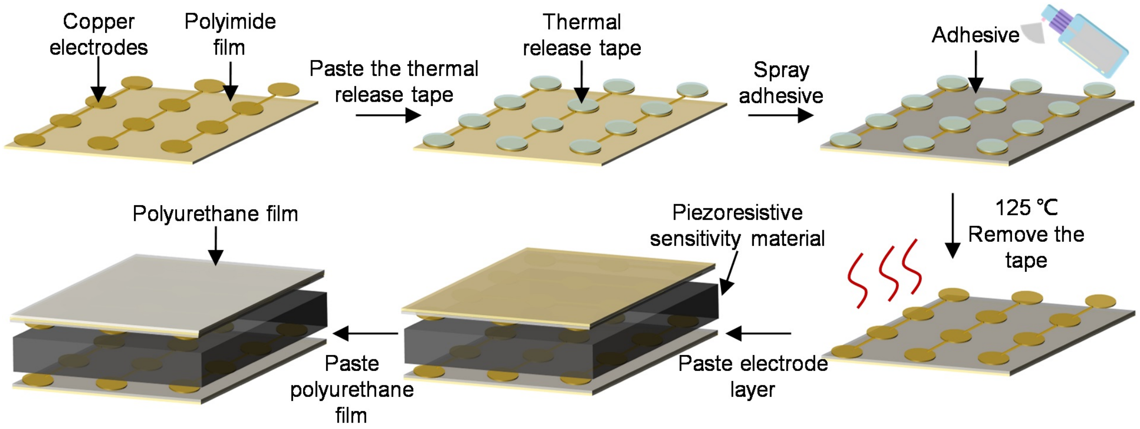


**Fig. S8.** The flowchart for manufacturing the flexible pressure sensor array.

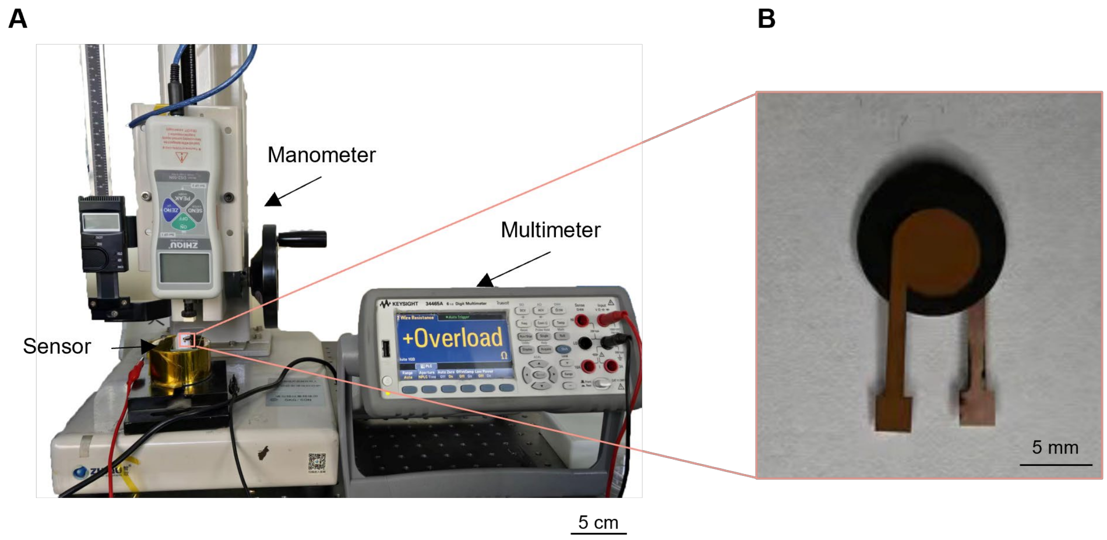


**Fig. S9.** (A) The platform for testing flexible the pressure sensor. (B) Images of a single pressure sensor.

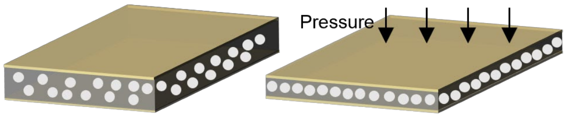


**Fig. S10.** Schematic diagram of the sensor mechanism.

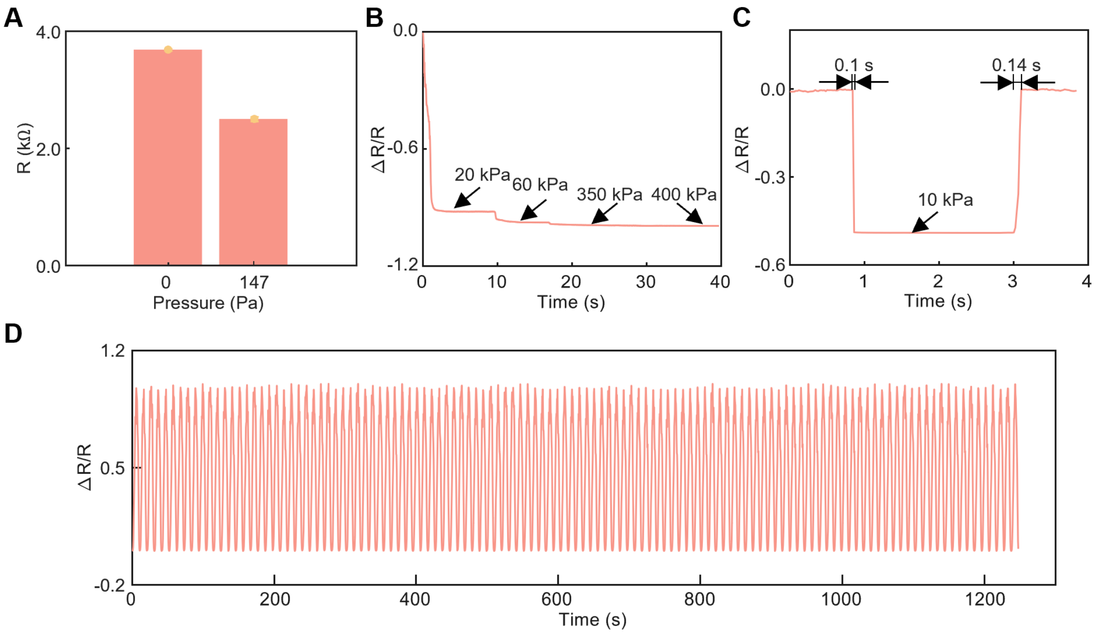


**Fig. S11.** Sensing performance characterization results. Experimental results of sensing detection limit (A), sensing range (B), sensing response/recovery time (C) and sensing dynamic performance (D).

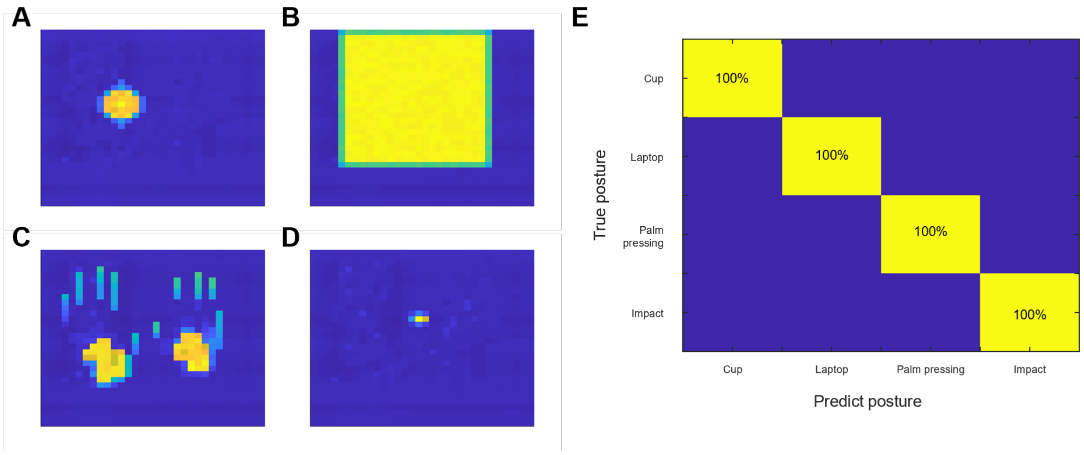


**Fig. S12.** Pressure response maps of the sensing array and the classification result under various tests. (A) Water cup and (B) laptop response maps to represent applications in robotic tactile sensing. (C) Palm pressing response map to illustrate human-machine interaction. (D) Impact response map to demonstrate the application in smart skin technology. (E) Classification confusion matrix of the four scenarios.

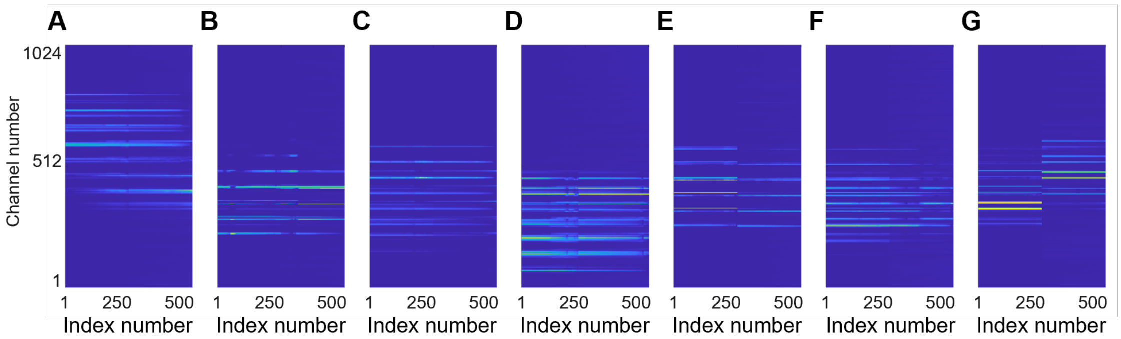


**Fig. S13.** The raw pressure data sample maps of seven typical sitting postures (from left to right, (A) lean backward, (B) lean left, (C) cross the right leg, (D) lounge, (E) lean right, (F) cross the left leg, and (G) sit straight).

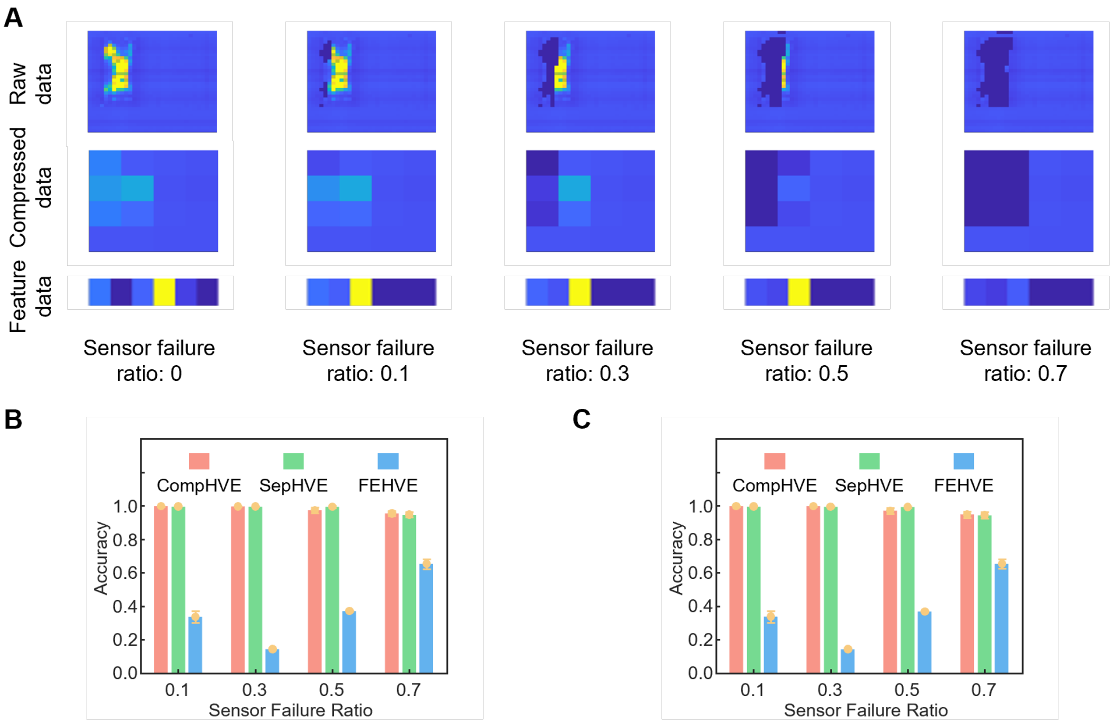


**Fig. S14.** (A) Response maps of the raw, compressed, and feature data under different sensor failure ratios (0.1, 0.3, 0.5, 0.7). Result graphs showing the impact of different sensor failure ratios on training set recognition accuracy (B) and testing set recognition accuracy (C).

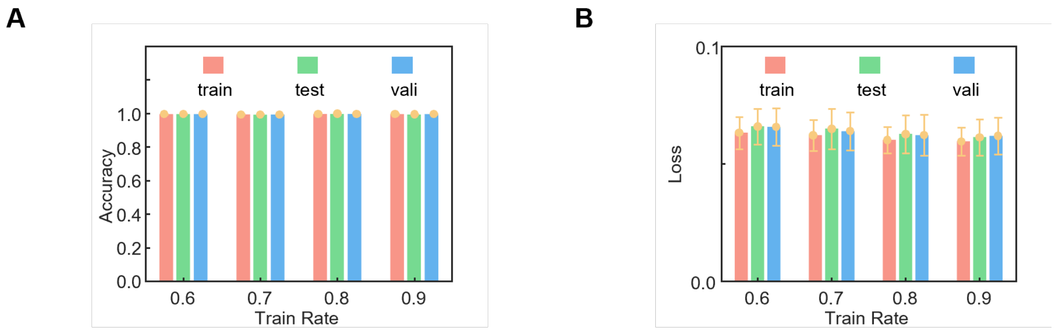


**Fig. S15.** Comparison of recognition accuracy (A) and loss changes (B) across the training, test, and validation data sets at various training rates.

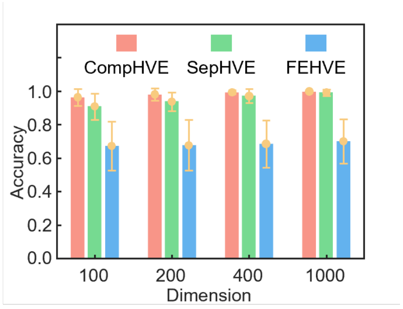


**Fig. S16.** The accuracy of three types of HDC models: the compression hypervectors (HVs) encoder (CompHVE), the separate HVs encoder (SepHVE), and the feature extraction HVs encoder (FEHVE).

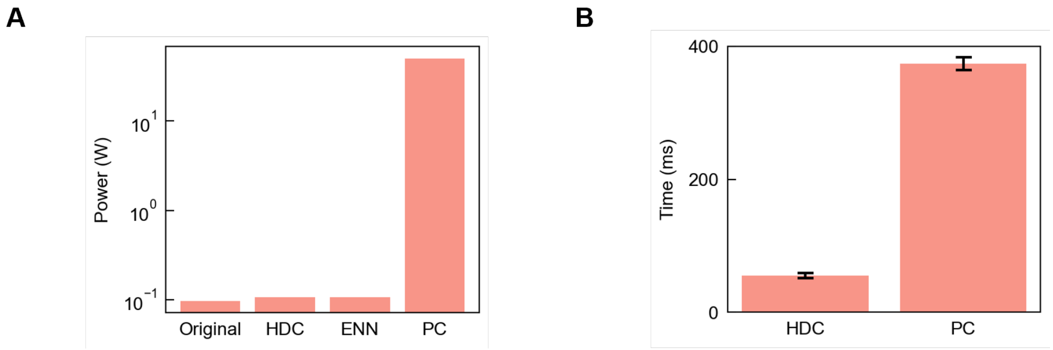


**Fig. S17.** Comparison of system power consumption (A) and system latency (B) across different methods.

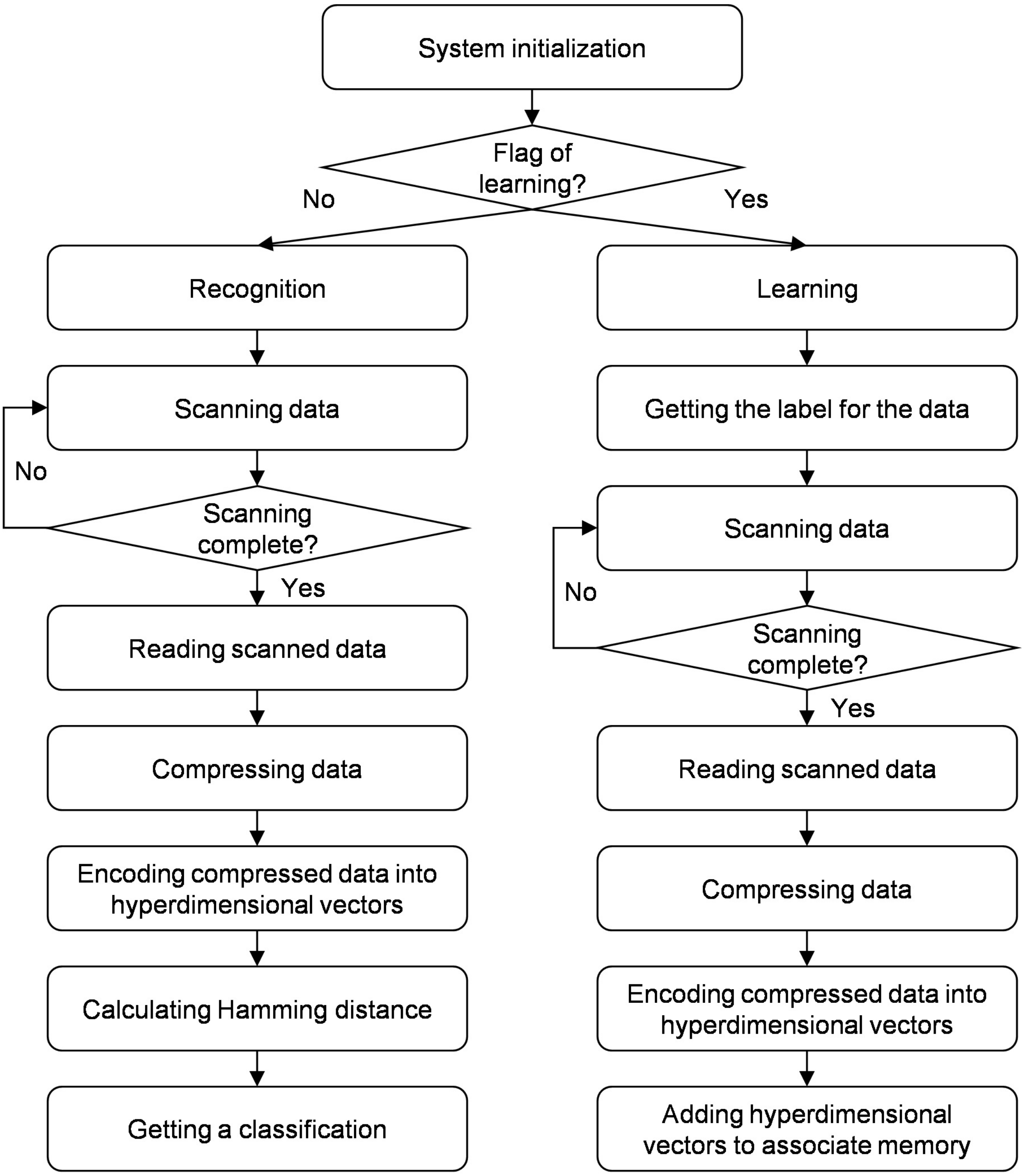


**Fig. S18.** The flowchart of on chip data analysis.

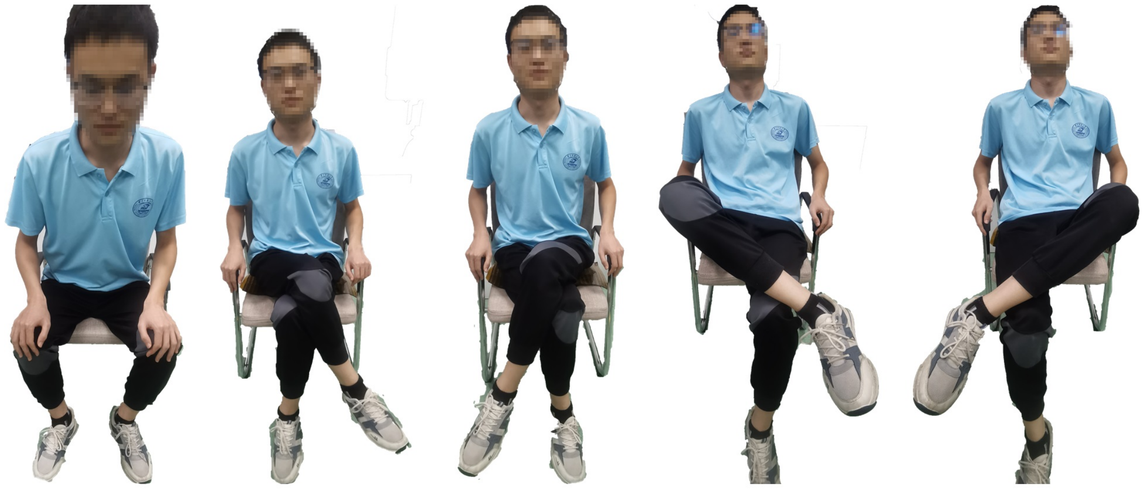


**Fig. S19.** Seven new sitting postures for in-sensor learning and recognition.

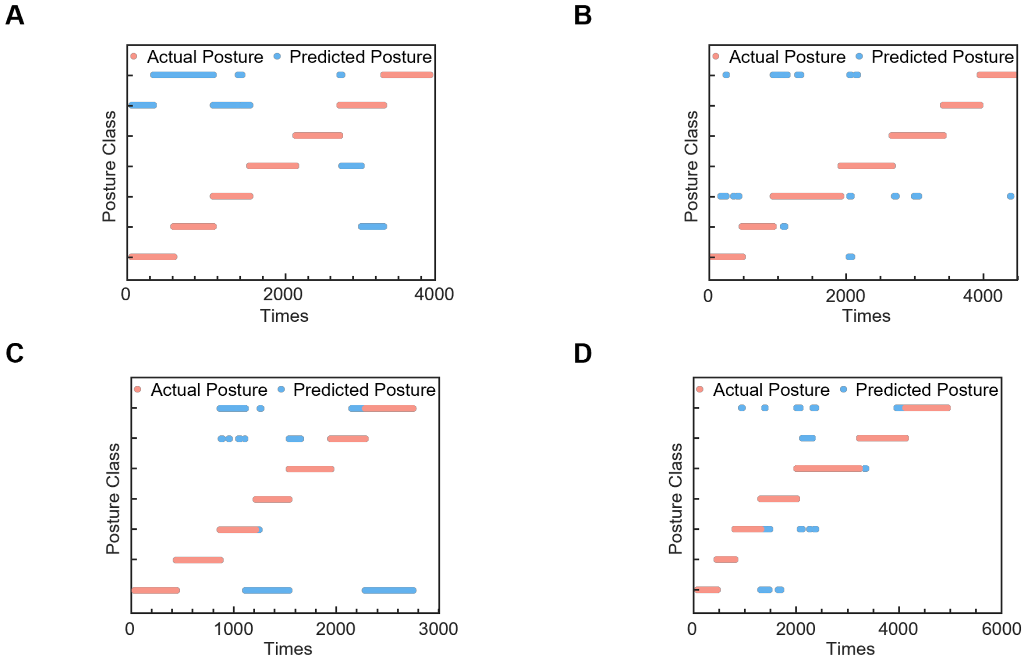


**Fig. S20.** Comparison of real-time predicted postures with actual postures. (A) Results of Participant No. 2 before in-sensor learning. (B) Results of Participant No. 2 after on-chip learning. (C) Results of Participant No. 3 before in-sensor learning. (D) Results of Participant No. 3 after in-sensor learning.
